# The role of LOXL2 induced by glucose metabolism-activated NF-κB in maintaining drug resistance through EMT and cancer stemness in gemcitabine-resistant PDAC

**DOI:** 10.1007/s00109-023-02369-6

**Published:** 2023-09-22

**Authors:** Yun Sun Lee, Hyung Sun Kim, Hyo Jung Kim, Hyeon Woong Kang, Da Eun Lee, Myeong Jin Kim, Woosol Chris Hong, Ju Hyun Kim, Minsoo Kim, Jae-Ho Cheong, Joon Seong Park

**Affiliations:** 1 Department of Surgery, Gangnam Severance Hospital, Yonsei University College of Medicine, Seoul, South Korea; 2 Department of Surgery, Yonsei University College of Medicine, Seoul, South Korea; 3 Department of Medical Science, Graduate School of Medical Science, Brain Korea 21 Project, Yonsei University College of Medicine, Seoul, South Korea

**Keywords:** Pancreatic cancer, LOXL2, Glucose metabolism, NF-κB, EMT, Cancer stem cell

## Abstract

**Abstract:**

Gemcitabine is considered a standard treatment for pancreatic cancer, but developing drug resistance greatly limits the effectiveness of chemotherapy and increases the rate of recurrence. Lysyl oxide-like 2 (LOXL2) is highly expressed in pancreatic cancer and is involved in carcinogenesis and EMT regulation. However, studies on the role of LOXL2 in drug resistance are limited. Here, we investigated the mechanism of LOXL2 induction and the effect of LOXL2 on EMT and CSC in gemcitabine-resistant pancreatic cancer. Glucose metabolism was activated in gemcitabine-resistant pancreatic cancer cells, and NF-κB signaling was regulated accordingly. Activated NF-κB directly induces transcription by binding to the promoters of LOXL2 and ZEB1. The EMT process was significantly inhibited by the coregulation of ZEB1 and LOXL2. In addition, LOXL2 inhibition reduced the expression of cancer stemness markers and stemness by regulating MAPK signaling activity. LOXL2 inhibits tumor growth of gemcitabine-resistant pancreatic cancer cells and increases the sensitivity to gemcitabine in mouse models.

**Key messages:**

- We identified a specific mechanism for inducing LOXL2 overexpression in gemcitabine-resistant pancreatic cancer. Taken together, our results suggest LOXL2 has an important regulatory role in maintaining gemcitabine resistance and may be an effective therapeutic target to treat pancreatic cancer.

**Supplementary Information:**

The online version contains supplementary material available at 10.1007/s00109-023-02369-6.

## Introduction

Pancreatic cancer ranks as the ninth leading cause of cancer-related mortality in Korea. Despite progress in techniques for detection and management, the 5-year survival rate only peaked at 13.9% [1]. It remains a deadly malignancy, with few symptoms evident before the disease reaches its advanced stage [2]. Overall, the low rate of early detection, rapid progression, drug resistance, and lack of proper therapy leads to a poor prognosis [3]. Pancreatic ductal adenocarcinomas (PDACs) account for 90% of pancreatic malignancies. The current standard of cure for resectable pancreatic cancer is surgery followed by adjuvant chemotherapy [4]. Although patients received successful surgical treatment, most will suffer from disease recurrence within a year [5]. Thus, the main considerations are undetected micrometastasis, which is limited to elimination by surgical resection and the development of chemical resistance.

Gemcitabine has been widely used as a first-line drug for advanced pancreatic cancer. Gemcitabine (dFdC) is a deoxycytidine nucleoside analog, which acts by inhibiting proliferation and blocking the cell cycle process at the G1/S phase boundary by suppressing DNA synthesis [6]. Although gemcitabine is considered effective among patients with advanced and metastatic pancreatic cancer, the development of chemoresistance severely limits the effectiveness of chemotherapy and increases the recurrence of control failure [7].

Malignant tumors known to have aberrant cellular metabolism exhibit the Warburg effect, in which large amounts of lactic acid are produced by aerobic glycolysis and reduced mitochondrial oxidative phosphorylation, resulting in growth benefit. The acidic tumor microenvironment created by excessive lactic acid production promotes the migration and invasion of tumor cells [8]. Furthermore, through increased glucose utilization because of overexpression of the glucose transporter (Glut), pancreatic cancer not only promotes tumorigenesis but also provides a favorable environment for drug resistance [9]. These persistent glycolytic changes allow cancer cells to adapt to a hypoxic environment, produce the biosynthetic building blocks necessary for cell proliferation, and acidify the local environment to promote tumor invasion. Subsequently, NADPH and glutathione are formed through a pentose phosphate shunt to increase resistance to oxidation [10]. Therefore, since the Warburg effect is considered a fundamental property of neoplasia, the ability of glucose transporter inhibition to enhance the sensitivity of pancreatic cancer cells to gemcitabine through inhibition of glucose uptake represents a therapeutically relevant strategy for cancer treatment [11].

Glucose metabolic enzymes and their products also exert multilevel control of nuclear factor kappa B (NF-κB) activity, creating a highly connected regulatory network [12]. The NF-κB signaling system, a key regulator of immunological processes, also affects a variety of metabolic changes associated with inflammation and the immune response. NF-κB–regulating signaling cascades, with NF-κB–mediated transcriptional events, control the metabolism at several levels. NF-κB modulates apical components of metabolic processes including metabolic hormones such as insulin and glucagon. The cellular master switches 5′ AMP-activated protein kinase and mTOR, as well as many metabolic enzymes and their respective regulators [13]. The role of NF-κB in glucose uptake reveals a comparably complex picture, because knockdown of p65 in mouse embryonic fibroblasts resulted in increased expression of GLUT3, whereas opposing observations described a positive role of NF-κB in its expression [14]. It was also proposed that glycolysis stimulates IKK/NF-κB activity, as revealed by reduced IKK activity in the presence of a glycolytic inhibitor and increased IKK activity after GLUT3 expression [15].

Lysyl oxidase like 2 (LOXL2) is an ECM-related enzyme catalyzing the formation of collagen crosslinks. Evidence has emerged to suggest that LOXL2 plays a role in the promotion of cancerous cell invasion, metastasis, and angiogenesis and malignant transformation of solid tumors [16]. Expression of LOXL2 in pancreatic cancer activates epithelial to mesenchymal transition (EMT)-induced signals and induces changes in EMT, affecting invasiveness. Several studies have attempted to determine the role of LOXL2 in gemcitabine resistance. Reduction of E2F5, one of the EMT-related transcription factors, by LOXL2, has been shown to increase sensitivity to gemcitabine [17]. Furthermore, the activity of the Lox family participated in the chemoresistance of pancreatic cancer by limiting the intratumoral distribution of gemcitabine [18].

Recently, it has been shown that the EMT process of cancer cells is associated with chemotherapy resistance [19]. According to other cancer studies, not all drug-resistant cells showed EMT, but they had undergone an EMT process and EMT cells had a selective growth advantage in the presence of the drug. It has been increasingly revealed that cancer drug resistance is frequently accompanied by EMT in diverse cancer. Furthermore, EMT signaling pathways and morphological and genetic changes by EMT were actually found to contribute to drug resistance [20]. In gemcitabine resistance in pancreatic cancer, ent1 and cnt3 are frequently upregulated in KPC mouse models with deleted snail or twist [21]. Furthermore, the Akt/GSK3β/snail 1 pathway was revealed to be the key signaling event leading to acquisition of gemcitabine resistance in pancreatic cancer [22]. However, the other underlying mechanisms of EMT in pancreatic cancer gemcitabine resistance are not fully studied.

Cancer stem cells (CSC) are reported to be responsible for tumor initiation as well as drug-resistant phenotypes, such as reduced rates of apoptosis, reduced rates of mitosis, and DNA damage [23–26]. Following CSC models, recent studies have identified the existence of three transcriptionally defined pancreatic cancer subtypes with different biological properties and subtype-specific drug responses [27, 28]. CD44 + CD24 + ESA + pancreatic cancer cells have been reported to exhibit significant resistance to GEM and radiation and contribute to pancreatic cancer recurrence and metastasis [29]. Therefore, targeting cancer stem cells by identifying the resistance mechanisms that support the expression of a resistant phenotype could be an effective method for treating gemcitabine-resistant pancreatic cancer.

## Materials and methods

### Patient selection

Patients diagnosed with pancreatic cancer at Gangnam Severance Hospital from 2018 to 2019 received pancreatic resection. All the patients had received adjuvant gemcitabine chemotherapy. Gemcitabine (1000 mg/m^2^) was infused over 30 min once a week for 3 out of 4 weeks. Each regimen was administered to the patients for 6 cycles. Patients who had not any recurrence for 1 year after receiving gemcitabine-based chemotherapy (6 cycles) were considered chemotherapy-sensitive, while the rest of these patients were regarded as chemotherapy-resistant (Table 1). The study protocol was approved by the Institutional Review Board at Gangnam Severance Hospital, Yonsei University of Korea (3–2014-0153) and complied with the Declaration of Helsinki. Informed consent was obtained from all participants.

**Table 1:** Summary of patient’s clinical characteristic

**Characteristic**	**Gemcitabine resistant (*n* = 16)**	**Gemcitabine sensitive (*n* = 6)**
**Age**		
Median	63	63
Range	47–77	37–73
**Sex**		
Male	6 (37.5%)	2 (33.3%)
Female	10 (62.5%)	4 (66.7%)
**Margin status**		
R0	14 (87.5%)	6 (100%)
R1	0 (0%)	0 (0%)
R2	2 (12.5%)	0 (0%)
**Tumor size**		
Mean ± SD, cm	3.57 (± 0.29)	3.28 (± 0.24)
**Operation name**		
PPPD	13 (81.3%)	5 (83.4%)
DP	3 (18.7%)	1 (16.6%)
**Cell differentiation**		
Well	2(12.5%)	3 (50%)
Moderate	13 (81.3%)	3 (50%)
Poor	1 (6.2%)	0 (0%)
**Lymphovascular invasion**		
No	3 (18.7%)	4 (66.7%)
Yes	6 (37.5%)	2 (33.3%)
Unknown	7 (43.8%)	0 (0%)
**Perineural invasion**		
No	1 (6.2%)	1 (16.6%)
Yes	5 (31.2%)	5 (83.4%)
Unknown	10 (62.6%)	0 (0%)
**N stage**		
N0	6 (37.4%)	3 (50%)
N1	10 (62.6%)	3 (50%)
N2	0 (0%)	0 (0%)
**AJCC stage 8**^**th**^		
lB	0 (0%)	1 (16.6%)
llA	3 (18.7%)	2 (33.3%)
llB	11 (68.8%)	3 (50.1%)
lll	2(12.5%)	0 (0%)
lV	0 (0%)	0 (0%)

*PPPD* pylorus preserving pancreaticoduodenectomy, *DP* distal pancreatectomy, *AJCC* American Joint Committee on Cancer

### Cell line construction

Mia PaCa-2 cells were obtained from the American Type Culture Collection (Manassas, VA, USA). Cells were cultured in DMEM (Biowest) supplemented with 10% FBS (Biowest) and 1% antibiotic–anti-mycotic reagent (Gibco, Waltham, MA, USA). The cells were incubated at 37 ℃ in a humidified atmosphere of 5% CO_2_. Gemcitabine-resistant cell lines Mia GR were constructed by exposing to increasing dosages of gemcitabine for 3 months, and then persistently culturing in medium containing 10 μM gemcitabine.

### RNA sequencing and data analysis

Total RNA was isolated using TRIzol reagent (Invitrogen). RNA quality was assessed by Agilent 2100 bioanalyzer using the RNA 6000 Nano Chip (Agilent Technologies, Amstelveen, The Netherlands), and RNA quantification was performed using ND-2000 Spectrophotometer (Thermo Inc., DE, USA). For control and test RNAs, library construction was performed using QuantSeq 3’ mRNA-Seq Library Prep Kit (Lexogen, Inc., Austria), according to the manufacturer’s instructions. High-throughput sequencing was performed as 75-bp single-end sequencing using NextSeq 500 (Illumina, Inc., USA). QuantSeq 3’ mRNA-Seq reads were aligned using Bowtie2 [30]. Differentially expressed genes were determined based on counts from unique and multiple alignments using coverage in Bedtools [31]. RC (Read Count) data were processed based on quantile normalization method using EdgeR within R [32] with Bioconductor [33]. Gene classification was based on searches performed using the DAVID (http://david.abcc.ncifcrf.gov/) and Medline databases (http://www.ncbi.nlm.nih.gov/).

### Reagents

2-Deoxy-D-glucose, WZB117, JSH-23, Adezmapimod, and SB202190 were purchased from MedChemExpress (NJ, USA). MG132 was purchased from Sigma-Aldrich (St. Louis, MO, USA).

### Cytotoxicity assay

Cells (3 × 10^3^ per well) were seeded into a 96-well cell culture plate. The next day cells were treated with gemcitabine for 72 h. After incubation, 10% WST-1 reagent (EZ-Cytox, Dogen, Korea) was placed into the wells after aspiration of growth medium. The absorbance of each well was measured at 450 nm using a VersaMax microplate reader (Molecular Devices, San Jose, CA, USA).

### Western blotting

Cells were harvested, washed with ice-cold PBS, and lysed using RIPA lysis buffer. Proteins (30 μg sample) were separated using SDS PAGE and transferred to nitrocellulose membranes, blocked in 5% skim milk, and incubated with the following primary antibodies (1:1000): anti-LOXL2, snail + slug, phospho-IKB, cell fractionation marker, and ZEB1 (Abcam); anti-hENT1, RRM1, DCK, IKB, phospho-NF-κB, NF-κB, vimentin, EPCAM, and KLF4 (Cell Signaling Technology); N-cadherin and E-cadherin (BD Biosciences, San Jose, CA, USA); c-myc, OCT4, and beta-actin (Santa Cruz biotechnology); and γ-tubulin (Sigma-Aldrich).

### RNA isolation and qPCR

After the indicated treatment, cells were collected and their RNA was isolated using TRIZOL Reagent^®^ (Sigma-Aldrich) according to the manufacturer’s instructions. Then, 0.2 µg total isolate RNA was analyzed via reverse transcriptase PCR using the One-Step RT-PCR Kit (iNtRON Biotechnology, Seongnam-si, Korea). First-strand cDNA synthesis was performed with 1 µg RNA as a template using the RT-qPCR cDNA Synthesis Kit (iNtRON Biotechnology), according to the manufacturer’s instructions. RT-qPCR was performed using the SYBR qPCR reaction mix (Applied Biosystems, Foster City, CA, USA). The primer sequences used in this study are listed in Supplementary Table S1. Relative mRNA expression level was calculated using the 2 − ΔΔCT method, with GAPDH as the reference gene.

### RNA interference

For gene knockdown, the following small interfering RNA (siRNA) was purchased from Bioneer (Daejeon, KR). Information about the sequence: siLOXL2 sense 5′-CAGUCUAUUAUAGUCACAU-3′, anti-sense 5′-augugacuauaauagacug-3′, siGLUT3 sense 5′-CCGCUGCUACUGGGUUUUA-3′, anti-sense 5′-UAAAACCCAGUAGCAGCGG-3′. Transfection was conducted using Lipofectamine RNAiMAX (Invitrogen) according to the manufacturer’s instructions. Cells were harvested and processed 48–72 h post-transfection.

### Glucose uptake

The 2-NBDG uptake assay was performed according to the manufacturer’s protocol (Cayman). Briefly, cells were seeded in plates for 48 h and incubated with 2-NBDG reagent and medium supplemented with glucose uptake enhancer for 30 min. The cells were then collected and washed in analysis buffer before flow cytometry analysis.

### Lactate production analysis

The lactate production assay was performed according to the manufacturer’s protocol (Dogen, Korea). In brief, assay buffer was added to the cells, and after grinding with a homogenizer, reaction mixture was added. After dispensing in a 96-well plate, the reaction was performed at 20–25 °C. Light was blocked for 30 min, followed by gentle shaking and measurement with a microplate reader (570 nm).

### Immunocytochemistry

Transfected cells were fixed with 3.7% paraformaldehyde for 10 min and permeabilized with 0.1% Triton X-100 for 30 min. Cells were washed with PBS three times and incubated with 1% bovine serum albumin for 1 h at room temperature. Primary antibodies (phosph-NF-κB, cell signaling) were incubated overnight at 4 ℃ (1:100 to 1:1000); then cells were incubated at room temperature with Alexa Fluor 488 or Alexa Fluor 555 conjugated secondary antibodies (1:1000). Cells were stained and mounted with DAPI (Abcam) and Fluoroshield mounting medium for 5 min. Immunostained cells were observed under Carl Zeiss LSM780 confocal microscope.

### Cell fractionation

Cellular fractionation was performed using a subcellular protein fractionation kit purchased from Abcam. The harvested cell pellets were resuspended in a cytoplasmic extraction buffer and incubated at 4 °C for 10 min with gentle mixing. The samples were agitated every 5 min and then centrifuged at 5000 × *g* for 5 min to collect the cytoplasmic fraction. Pellets were resuspended, incubated in mitochondrial fraction buffer at 4 °C for 10 min, and centrifuged at 5000 × *g* for 5 min to obtain the nuclear fraction. The resuspended cytosol- and mitochondria-depleted remainder of cells contained nuclei and thus represent the nuclear fraction. Samples were incubated with SDS-PAGE Sample Buffer for 10 min in 60 °C, before proceeding with western blot analysis.

### Chromatin Immunoprecipitation (ChIP)

ChIP assays were performed with the SimpleChIP^®^ Enzymatic Chromatin IP Kit (cell signaling) following the manufacturer’s instructions. A total of 5 × 10^6^ Mia-Paca2 cells were cross-linked with 1% formaldehyde at room temperature for 10 min. Sonication was performed on ice (power: 0.5W, time: 2 s ON/15 s OFF, total time: 16 s × 3 rounds) to obtain 200–1000 bp DNA fragments. The chromatin was then immunoprecipitated with anti-IgG antibody (Cell signaling) and anti- NF-κB, ZEB1 antibody. After reverse cross-linking and DNA isolation and purification, DNA from input (1:100 diluted) or immunoprecipitated samples were assayed by quantitative PCR (Supplementary Table S2).

### Wound healing assay and invasion assay

For the scratch wound migration assay, transfected cells were plated at 1.2–1.4 × 10^4^ on a 24-well plate. Wound scratches were made 24 h after plating. Microscope images of migrated cells were taken every 24 h.

For invasion assays, 8-µm pore size Transwell system (Corning Inc.) was coated with Matrigel (1:50, Corning) for 1 h at room temperature. Subsequently, 2 × 10^4^ transfected cells were seeded on the apical side of the Transwell chamber (24-well insert) in serum-free media and growth media was added to the basal compartment. The cells were allowed to invade for 24 h. The remaining cells at the top of the chamber were gently scraped off using wetted cotton swabs. The cells that had invaded the basal side were fixed in methanol for 10 min, stained with 0.2% crystal violet, and then washed multiple times with 3’DW. Migration and invasion assays were performed in triplicate and repeated three times independently.

### Spheroid formation assay

Cell lines were grown in their standard culture conditions, harvested, and dissociated into single cell suspensions for spheroid generation. Cells were seeded in their optimal conditions in ultra-low attachment (ULA) round-bottom 96-well plates (Corning, 7007) in a volume of 200 µL/well in universal neurosphere culture medium (DMEM/F-12, 2% B-27 supplement, 1% 2 µg/mL Heparin, 20 ng/mL EGF, and 10 ng/mL bFGF, made fresh weekly). Plates were incubated at 37 °C in 5% CO_2_ and high humidity and spheroids were maintained by performing 50% medium replenishments every 3–4 days.

### 3D Culture

For 3D cell culture, the Cellrix^®^ 3D Culture System (Cellrix, Korea) was used according to the manufacturer’s instructions. The casting tray was covered with the mold and then allowed to stand for 30 min at 4 ℃. The cells were resuspended in Bio-Gel solution. One hundred twenty microliters of cell mixed Bio-Gel solution was dispensed into each well of casting gel. Using a spatula, Bio-Gel was scooped from the casting gel and placed into separate wells of 24-well plate containing media.

### Flow cytometry

Cells were stained with the following fluorochrome-conjugated monoclonal antibodies: anti-human FITC-CD44 and BV421-CD326 (eBioscience, San Diego, CA, USA). Live cells were classified by propidium iodide (BD Biosciences) staining. Stained cells were analyzed using BD FACSCanto II Cell Analyzer (BD Biosciences). FlowJo software (BD Biosciences) was used for compensation and data analysis.

### Knockdown stable cell lines

Five small hairpin ribonucleic acid (shRNA) LOXL2 sequences in lentiviral vector (pLKO.1-Puro) and control shRNA were purchased from Sigma-Aldrich. Lentiviruses were produced and transfected to Hek293 cells using Lipofectamine 3000 (Invitrogen). Mia-Paca2 cells were infected with purified viral particles, stably selected, and maintained with 2 μg/mL puromycin treatment.

### Mouse tumor models

All animal studies were conducted with a protocol proposal approved by the animal ethics committee of Yonsei University College of Medicine (approval #2019–0104). BALB/c nude mice (age, 6 weeks, male) were purchased from Oriente Bio. The pancreas was surgically exposed by abdominal excision under anesthesia with intraperitoneal injection (i.p.) of Alfaxan (25 mg/kg). Human pancreatic cancer cells were subcutaneously injected using a 30 G needle (BD bioscience). 4 × 10^6^ Mia-Paca2 cells were mixed with serum-free DMEM and Matrigel (1:1) and injected with 120 μL. After the operation, the mice were warmed and monitored until conscious and then placed in HEPA-filtered cages with food and water. After an indicated number of weeks, the mice were sacrificed and examined for tumor spread using macroscopic and microscopic observations by hematoxylin and eosin (H&E) staining.

### Statistical analysis

Statistical analysis was performed using GraphPad Prism version 8.01 software (GraphPad Software, La Jolla, CA, USA). Unpaired *t*-test was performed for the analysis of cell proliferation, western blotting, and qPCR data. The wound healing assay, invasion assay, and FACS data were analyzed by a two-way ANOVA. Differences were considered statistically significant at * *p* < 0.05 and ** *p* ≤ 0.01.

## Results

### LOXL2 is upregulated in gemcitabine-resistant pancreatic cancer cell lines

**Fig. 1:**
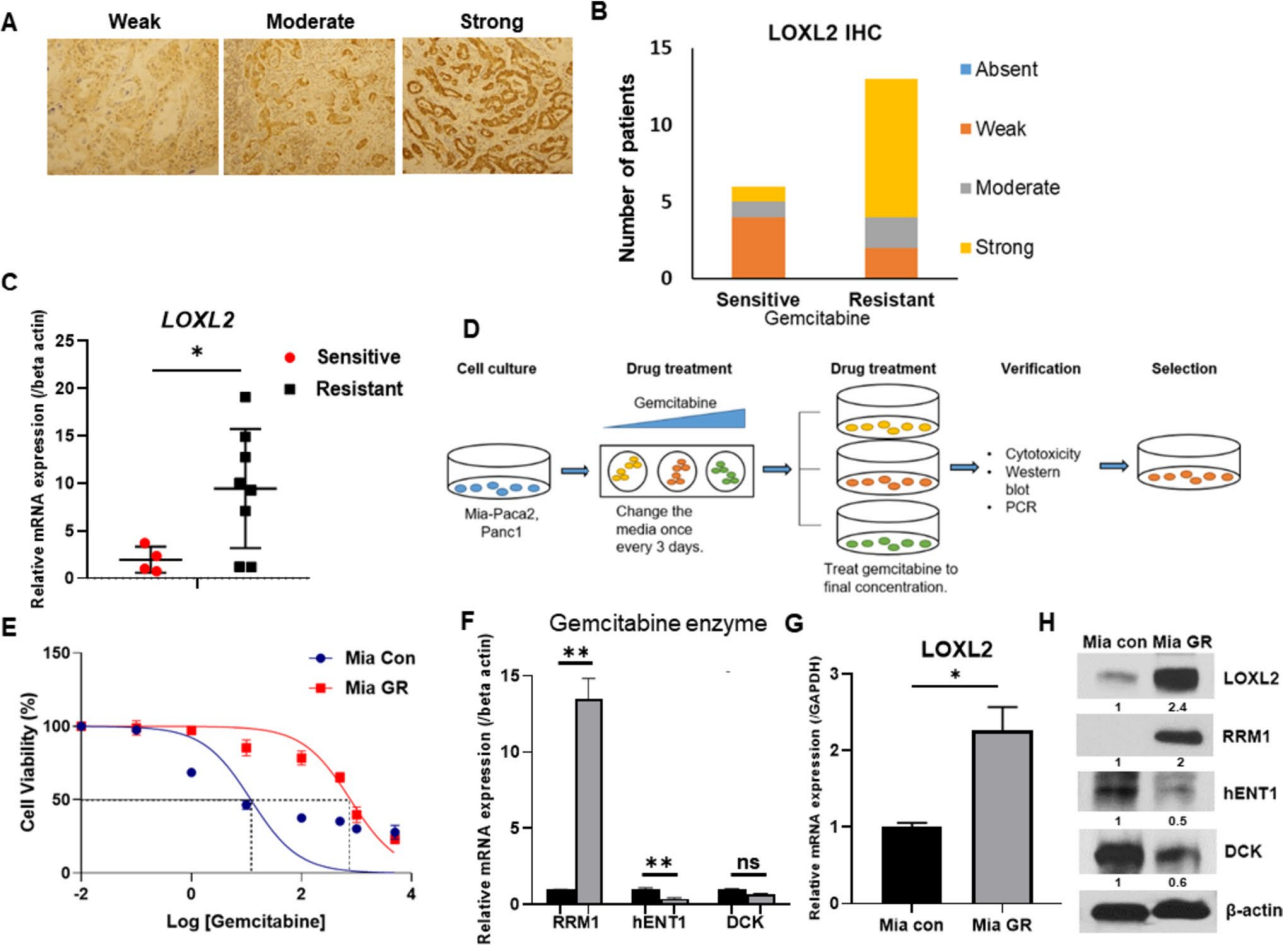
LOXL2 is highly expressed in patients with gemcitabine-resistant pancreatic cancer and gemcitabine-resistant pancreatic cancer cells. **A** Immunohistochemical staining of LOXL2 in pancreatic cancer tissues. **B** Bar graphs for LOXL2 levels in tissues between the resistant and sensitive groups. **C** Dot plot of LOXL2 gene expression level in purified mRNA from pancreatic cancer patient tissue. **D** Schematic showing the establishment of a gemcitabine-resistant cell line. **E** WST-1 assay of parental Miapaca2 (Mia Con) and gemcitabine-resistant (Mia GR) cells exposed to gemcitabine at different concentrations for 72 h. **F** and **G** Relative mRNA levels of the indicated genes in Miapaca2 and Mia GR cells. **H** The protein expression of LOXL2, RRM1, hENT1, and DCK was compared between Mia Con and Mia GR cells using western blot analysis. All western blot experiments were performed at least three times

Patients who underwent pancreatic cancer surgery and received gemcitabine adjuvant chemotherapy were classified into resistant and sensitive groups based on recurrence within 1 year (Table 1). Following immunohistochemical staining with LOXL2, the resistant group showed a higher ratio at strong intensity compared to the sensitive group (Fig. 1A, B). Total RNA was isolated from tissues of patients with pancreatic cancer and the difference in LOXL2 expression was compared. Therefore, it was possible to confirm the high LOXL2 expression in the resistant patient group (Fig. 1C). To elucidate the role of LOXL2 in vitro, we established gemcitabine-resistant Mia-Paca2 (Mia GR) by continuous exposure to gemcitabine in stepwise increments from each parental cell line (Mia Con) (Fig. 1D). When the final treatment concentration of gemcitabine-resistant cells was 10 μM, the most consistent resistance was confirmed. Gemcitabine-resistant cell lines demonstrated a clear increase in gemcitabine tolerance compared with parental cell lines, with half-maximal inhibitory concentration (IC50) of gemcitabine increasing from 12.03 to 774.5 μM (Fig. 1E). We identified known factors related to gemcitabine resistance in the prepared Mia GR through qPCR [34]. Several ATP-binding cassette (ABC) transporters and ALDH were confirmed to increase in Mia GR compared to Mia Con (Fig. S1A, B). In addition, it was found that among the factors related to gemcitabine metabolism, RRM1 increased and, conversely, hENT and DCK decreased in Mia GR (Fig. 1F) [35]. By western blotting, gemcitabine metabolism-related proteins were shown to increase (Fig. 1H). Therefore, it could be confirmed that Mia GR was generated accurately and consistently as a cell model to study the mechanism of gemcitabine resistance. LOXL2 mRNA and protein expression were confirmed to increase significantly compared to Mia Con (Fig. 1G, H). In addition, overexpression of LOXL2 was confirmed when a gemcitabine-resistant cell line was created using the PANC1 cell line (Fig. S1C, E). We could hypothesize that LOXL2 was overexpressed in tissues and cells of patients with gemcitabine-resistant pancreatic cancer based on confirmed results and that it would play an important role in the resistance-related mechanism.

### Overview of mRNA expression and enrichment analyses of gemcitabine-resistant pancreatic cancer cell lines

**Fig. 2:**
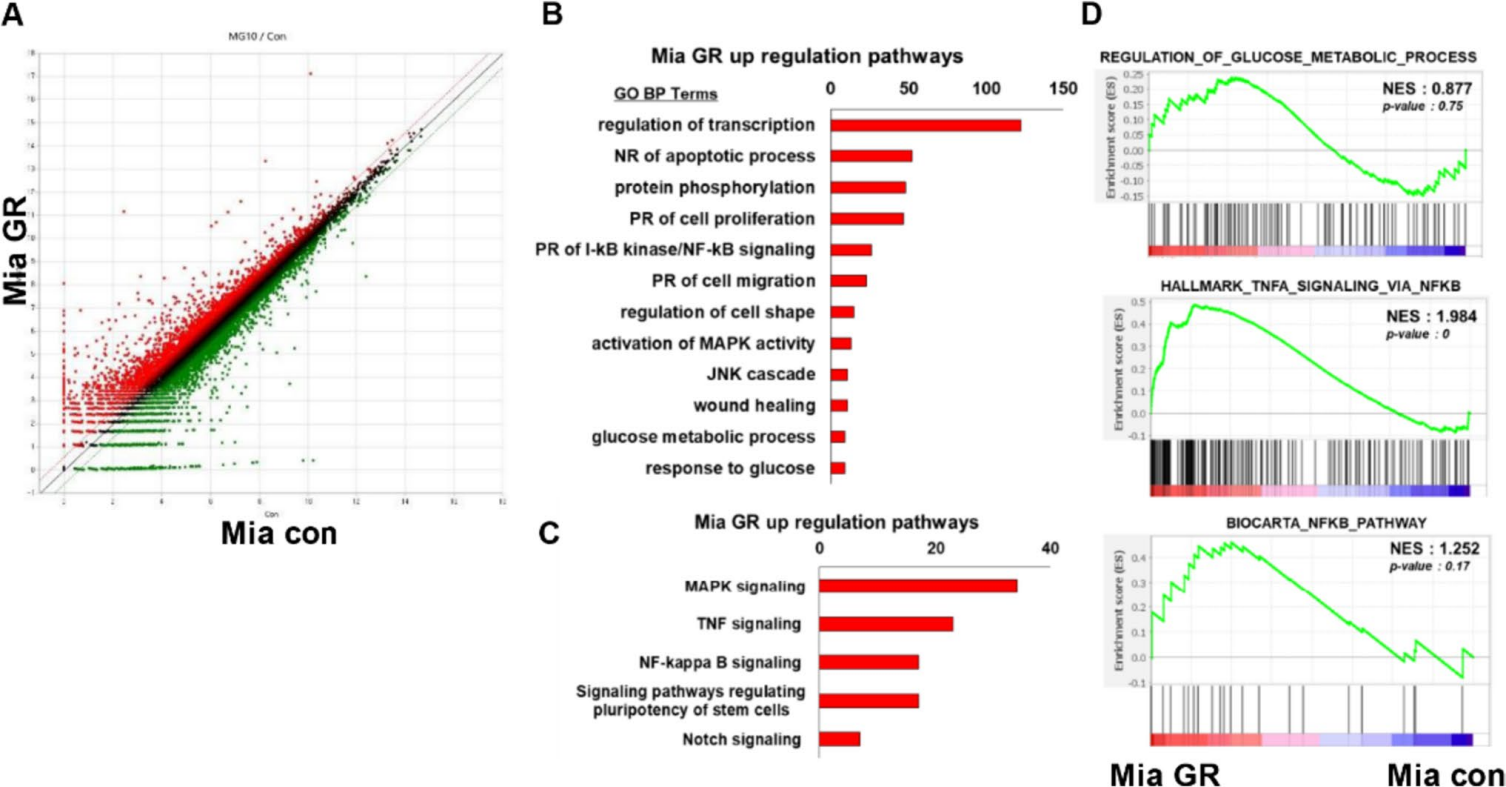
mRNA seq analysis of gemcitabine-resistant pancreatic cancer cells. **A** Scatter plots indicate the distribution of genes from Mia Con and Mia GR cells. **B** DAVID-based gene ontology and **C** KEGG pathway analysis of mRNA seq results from the Mia GR cells. **D** Gene Set Enrichment Analysis (GSEA) determined significant enrichment for glucose metabolic process, TNF signaling via NF-κB and NF-κB pathway in Mia GR and Mia Con cells.

Given the different phenotypes between Mia GR and Mia Con, we next analyzed the mRNA profiles between these cells using RNA sequencing. In addition, by using Mia GR transfected with siLOXL2, we attempted to check the mRNA profile change according to LOXL2. From the scatterplot data, we identified many genes with distribution differences between Mia Con and Mia GR cells (Fig. 2A). Using the DAVID analysis tool, the gene that showed high expression in the Mia GR group belonged to the gene ontology and the pathway through KEGG analysis was confirmed (Fig. 2B). Genes were included in biological processes expected to be associated with chemical resistance, such as negative regulation of apoptotic processes, positive regulation of cell proliferation, positive regulation of migration, and wound healing. Processes related to glucose metabolism were also included. The pathway was confirmed to include MAPK-JNK, TNF, and NF-κB signaling processes, and genes with high expression in Mia GR are included in stem-related signaling processes. In Mia GR transfected with siLOXL2, genes involved in apoptosis, proliferation, ECM, and migration-related biological processes were present. Through KEGG analysis, it was confirmed that these genes belong to the PI3K-Akt, Focal adhesive, MAPK, and TNF signaling pathways (Fig. 2B). From the gene set enrichment analysis (GSEA) results, the gene ontology to which the genes increased in Mia GR belonged was confirmed (Fig. 2C). Among them, glucose metabolism and NF-κB signaling were expected to be associated with increased LOXL2 expression.

### Glucose metabolism is activated in gemcitabine-resistant pancreatic cancer

**Fig. 3:**
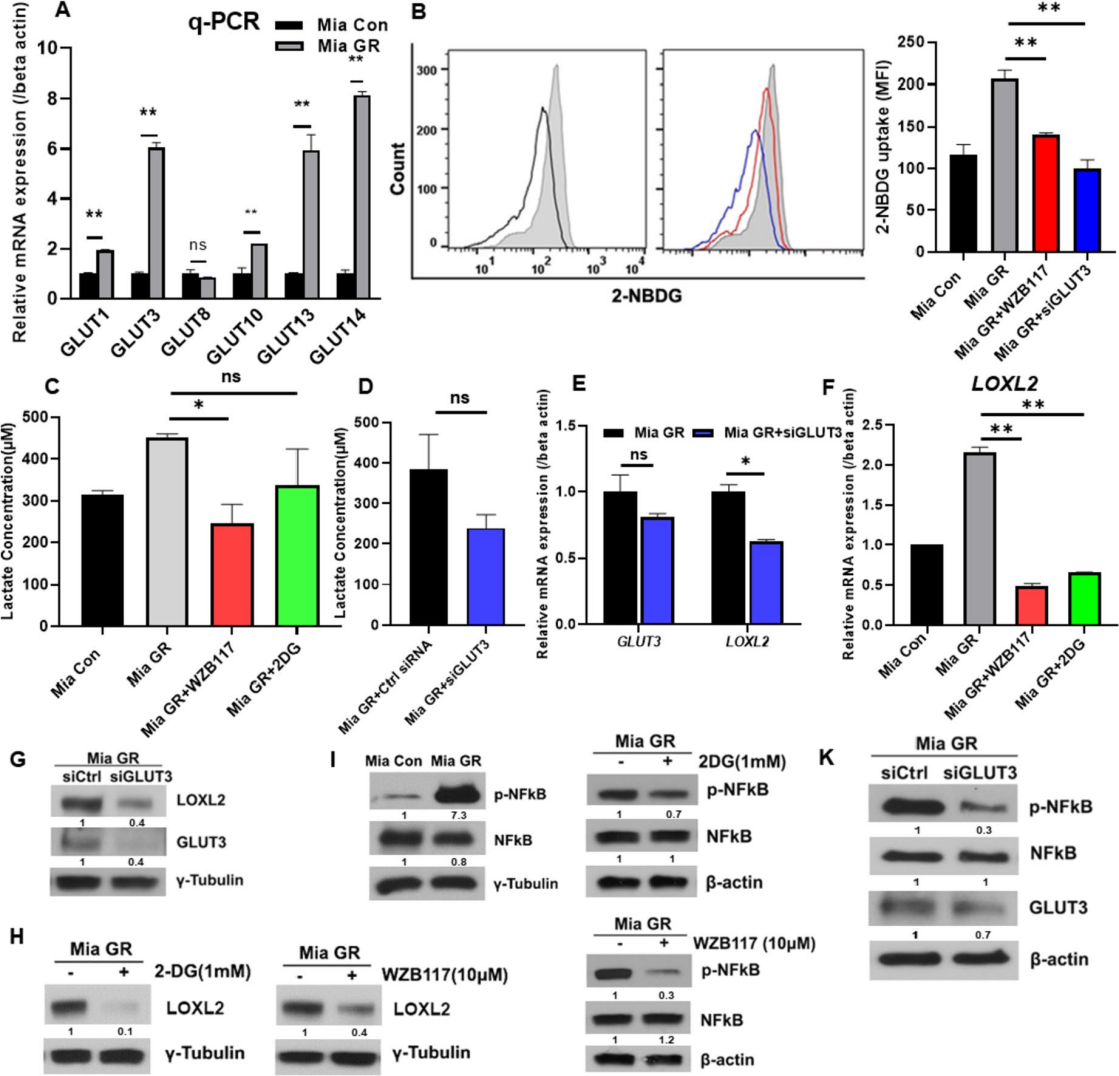
Regulation of LOXL2 expression by NF-kB activation through glucose metabolism in gemcitabine-resistant pancreatic cancer cells. **A** The mRNA levels of the GLUT genes were quantified by qPCR in Mia con and Mia GR. **B** Comparison of 2-NBDG uptake between Mia Con and Mia GR (no treatment, WZB117 treatment, siGLUT3 transfection) by flow cytometry histograms and graphs. **C** Comparison of lactate production between Mia Con and Mia GR (no treatment, WZB117 or 2DG treatment, siGLUT3 transfection (**D**)). **E** The mRNA levels of GLUT3 and LOXL2 in Mia GR and Mia GR transfected with siGLUT3. **F** The mRNA levels of LOXL2 in Mia Con and Mia GR (no treatment, WZB117 or 2DG treatment). **G** and **K** Mia GR cells were transfected with the control or GLUT3 siRNA. GLUT3, LOXL2 G, and p-NF-κB, NF-κB, and GLUT3 K were analyzed by immunoblotting. **H** and **J** 2-DG and WZB117 were treated with Mia GR cells and cell lysates were immunoblotted. **I** The phospho NF-κB and NF-κB protein expression in Mia Con and Mia GR were processed by immunoblotting

As seen from the mRNA seq results, genes more highly expressed in Mia GR compared to Mia Con belong to glucose metabolism. The Mia GR group exhibited a high expression of glut, which is involved in glucose uptake (Fig. 3A). Glucose uptake was compared using 2-NBDG, and a substantial increase in Mia GR was confirmed (Fig. 3B). Glucose uptake was reduced when Mia GR was treated with the glut inhibitor WZB117 and GLUT siRNA (Fig. 3B). Production of lactic acid, a product of glucose utilization, was found to increase in the MIA GR group (Fig. 3C, D). Previously, treatment with siGLUT3 and WZB117 revealed a decrease in glucose uptake and downregulated LOXL2 expression, and it was confirmed through qPCR and western blotting that LOXL2 expression was reduced even after treatment with 2DG, a glycolysis inhibitor (Fig. 3E, H). Therefore, it was concluded that increased glucose metabolism in gemcitabine-resistant cells induces and regulates LOXL2 expression.

### NF-κB signaling is regulated by glucose metabolism in gemcitabine-resistant cell lines

One of the signaling pathways activated in chemoresistance, NF-κB, was highly regulated in mRNA seq analysis; therefore, we attempted to confirm the association with glucose metabolism. By western blot analysis, we showed that phospho-NF-κB expression was increased in Mia GR (Fig. 3I). In contrast, treatment with the GLUT inhibitors WZB117 and 2DG reduced NF-κB phosphorylation (Fig. 3J, K). Treatment with siGLUT3 was found to reduce phosphor-NF-κB expression of phosphor-NF-κB and decrease nuclear translocation (Fig. S2A, B). Therefore, it was confirmed that glucose metabolism regulates NF-κB activation.

### Activated NF-κB binds to the LOXL2 promoter and transcriptionally regulates overexpression

**Fig. 4:**
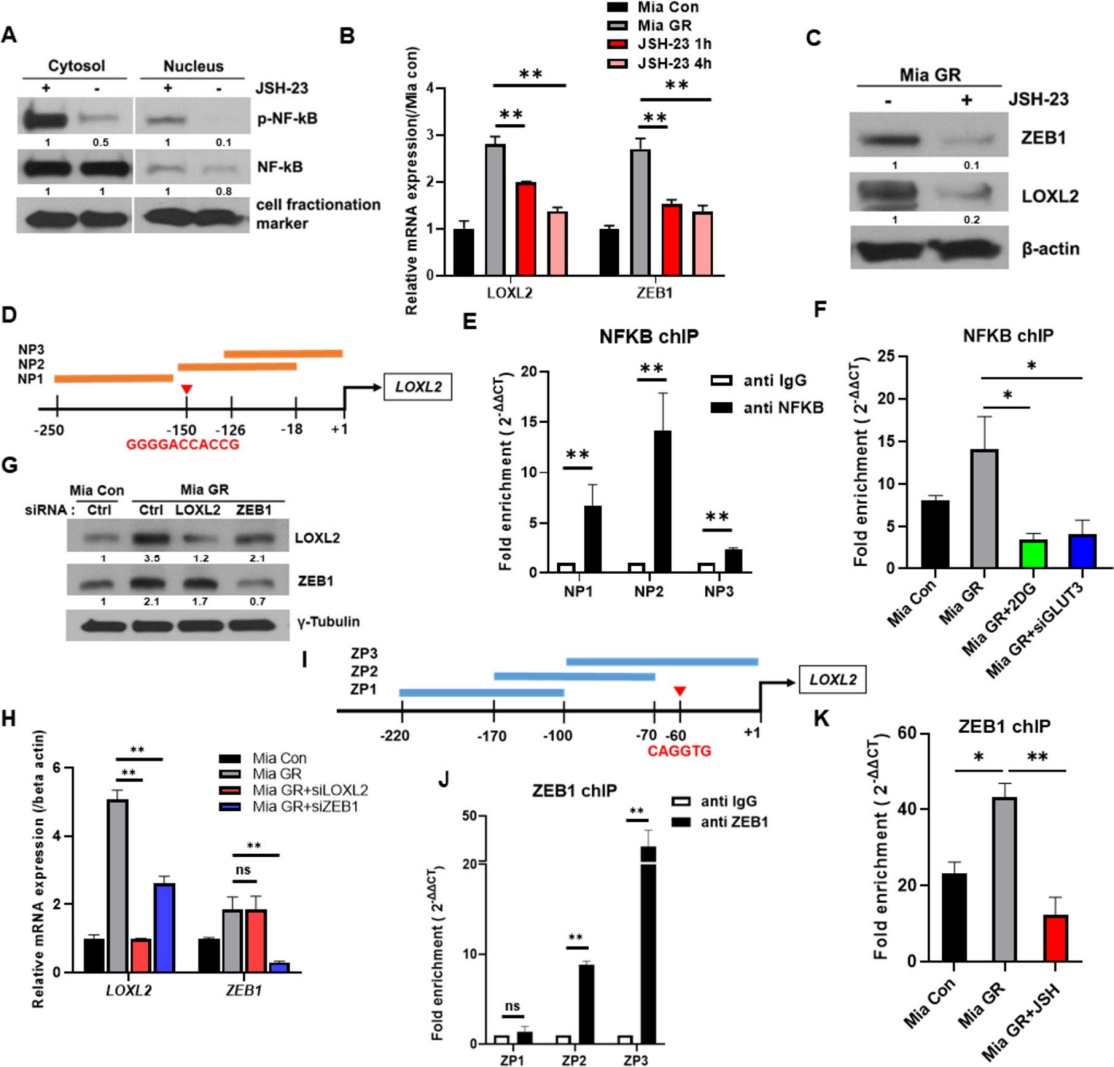
NF-kB directly regulates LOXL2 expression or indirectly through ZEB1. **A** Immunoblotting for phospho NF-κB and NF-κB using nuclear and cytoplasmic fractions prepared from Mia GR cells after treatment with JSH-23 for 4 h. Relative mRNA expression (**B**), and protein expression (**C**), of LOXL2 and ZEB1 in Mia Con, Mia GR (no treatment, JSH-23 1 h or 4 h treatment) cells. **D** Scheme for the expected NF-kB binding site on the LOXL2 gene promoter. **E** ChIP fold enrichment of LOXL2 DNA fragments (NP1, NP2, NP3) by chIP-qPCR. **F** Changes in chIP fold enrichment with NF-kB in the NP2 region on the LOXL2 gene promoter in Mia Con and Mia GR cells (no treatment, 2DG treatment, and siGLUT3 transfection) (**G**), Mia GR cell transfected with LOXL2 and ZEB1 siRNA. Knockdown efficiencies were evaluated by immunoblotting using LOXL2 and ZEB1 antibodies. **H** Relative gene expression of LOXL2 and ZEB1 confirmed by qPCR in Mia GR transfected with siRNA. **I** Scheme for the expected ZEB1 binding site on the LOXL2 gene promoter. **J** ChIP fold enrichment of LOXL2 DNA fragments (ZP1, ZP2, ZP3) by chIP-qPCR. **K** Changes in chIP fold enrichment with NF-kB in the ZP3 region on the LOXL2 gene promoter in Mia Con and Mia GR cells (no treatment, JSH-23 4 h treatment)

NF-κB is phosphorylated and regulates the transcription of several genes. First, JSH-23, known as NF-κB inhibitor, significantly inhibits the nuclear translocation of phosphorylated NF-κB in gemcitabine-resistant cells (Fig. 4A). It was confirmed that the expression of LOXL2 and ZEB1 was regulated with time when Mia GR was administered to JSH-23, an inhibitor of NF-κB (Fig. 4B, C). To determine whether NF-κB transcriptionally regulates LOXL2 expression, we analyzed the NF-κB binding region within the LOXL2 promoter using JASPAR (Fig. 4D). We could predict the 5′-GGGGACCACCG-3′ region and confirm that a high binding was formed in the vicinity, using chIP (Fig. 4E). Furthermore, treatment with 2DG, a glycolysis inhibitor, was shown to decrease binding to the expected NP3 region (Fig. 4F).

ZEB1, like LOXL2, is a representative factor that regulates EMT and is known to be directly activated by NF-κB [36]. We treated each siRNA to confirm the regulatory relationship between LOXL2 and ZEB1. By western blotting and qPCR, it was possible to confirm that LOXL2 was regulated by ZEB1 (Fig. 4G, H). Based on the JASPAR program, the LOXL2 promoter binding region of ZEB1 was specified and chIP was performed (Fig. 4I). High binding was confirmed in the ZP3 region (Fig. 4J). JSH-23 significantly inhibited the binding of ZEB1 to the LOXL2 promoter region (Fig. 4K). Therefore, it was concluded that LOXL2 overexpression caused by NF-κB activation in gemcitabine-resistant cells occurred either directly or directly through ZEB1.

### LOXL2 and ZEB1 are together involved in the regulation of EMT in gemcitabine-resistant pancreatic cancer

**Fig. 5:**
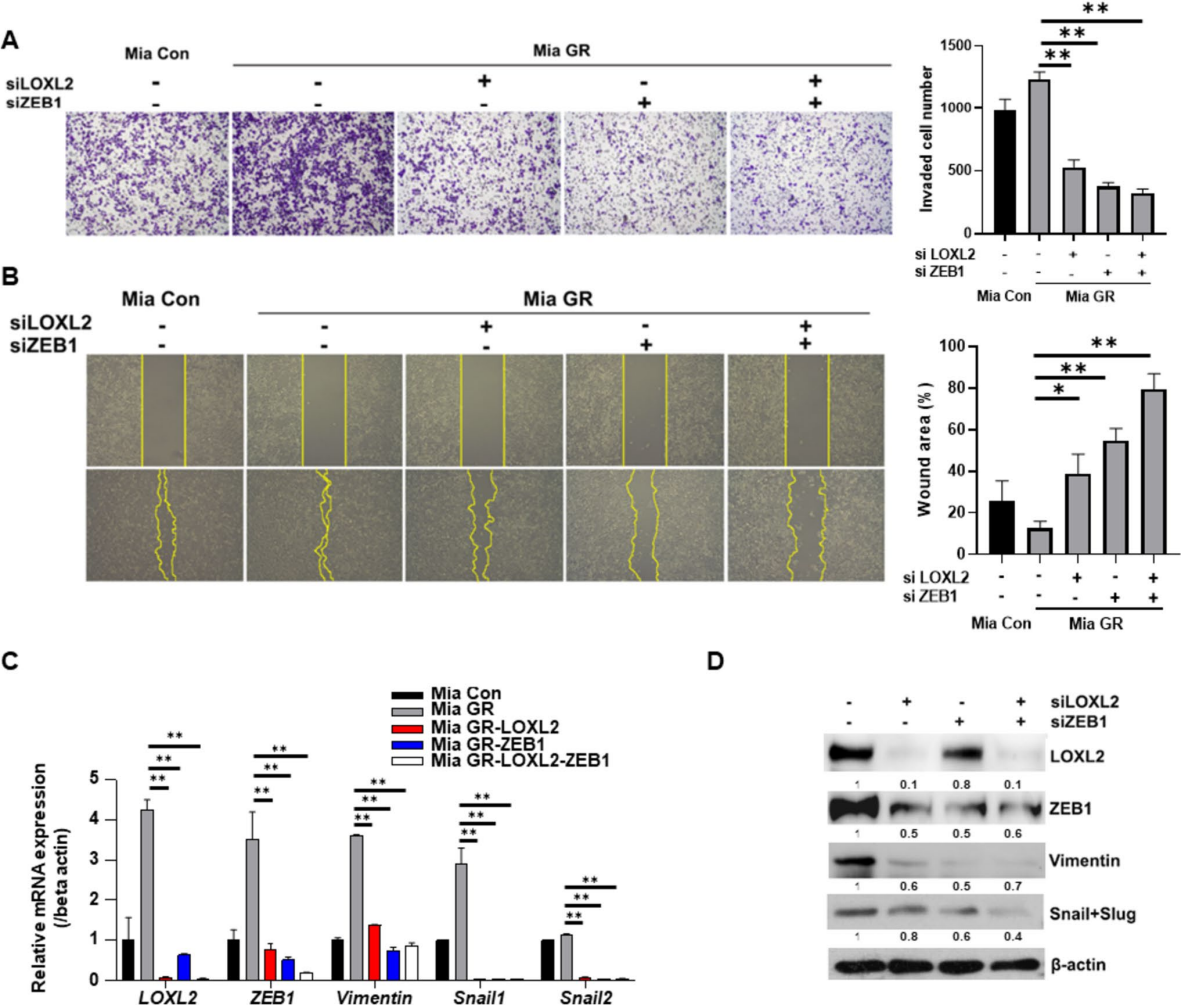
ZEB1-LOXL2 regulates the EMT process in gemcitabine-resistant cell lines. **A** Representative photographs and bar graph of the number of invaded cells that pass through the Matrigel-coated Transwell invasion assay. Mia GR transfected with LOXL2 and ZEB1 siRNAs. **B** The migration of cells was measured with wound healing assay. The quantitative evaluation and statistical analysis of wound closure percentage in wound healing assay were measured by Image J software. **C** and **D** The EMT marker expression of siRNA transfected Mia GR was analyzed by qPCR (**C**), and immunoblotting (**D**)

Mia GR was morphologically closer to mesenchymal cells than Mia Con, and migration-related genes were highly regulated in mRNA seq. Therefore, we attempted to confirm the change in invasiveness and migration ability of LOXL2-ZEB1. Based on the invasion assay, the invasiveness of LOXL2-ZEB1 knockdown cells was significantly reduced and a decrease in migration activity was confirmed by wound analysis (Fig. 5A, B). In addition, it was verified through qPCR that the expression of EMT-related genes and protein markers was also reduced (Fig. 5C, D). Therefore, the high EMT process in gemcitabine-resistant pancreatic cancer was confirmed to be regulated by the ZEB1-LOXL2 axis that contributes to maintaining chemoresistant characteristics.

### MAPK activation by LOXL2 regulates cancer stemness of EPCAM-dependent gemcitabine-resistant pancreatic cancer

**Fig. 6:**
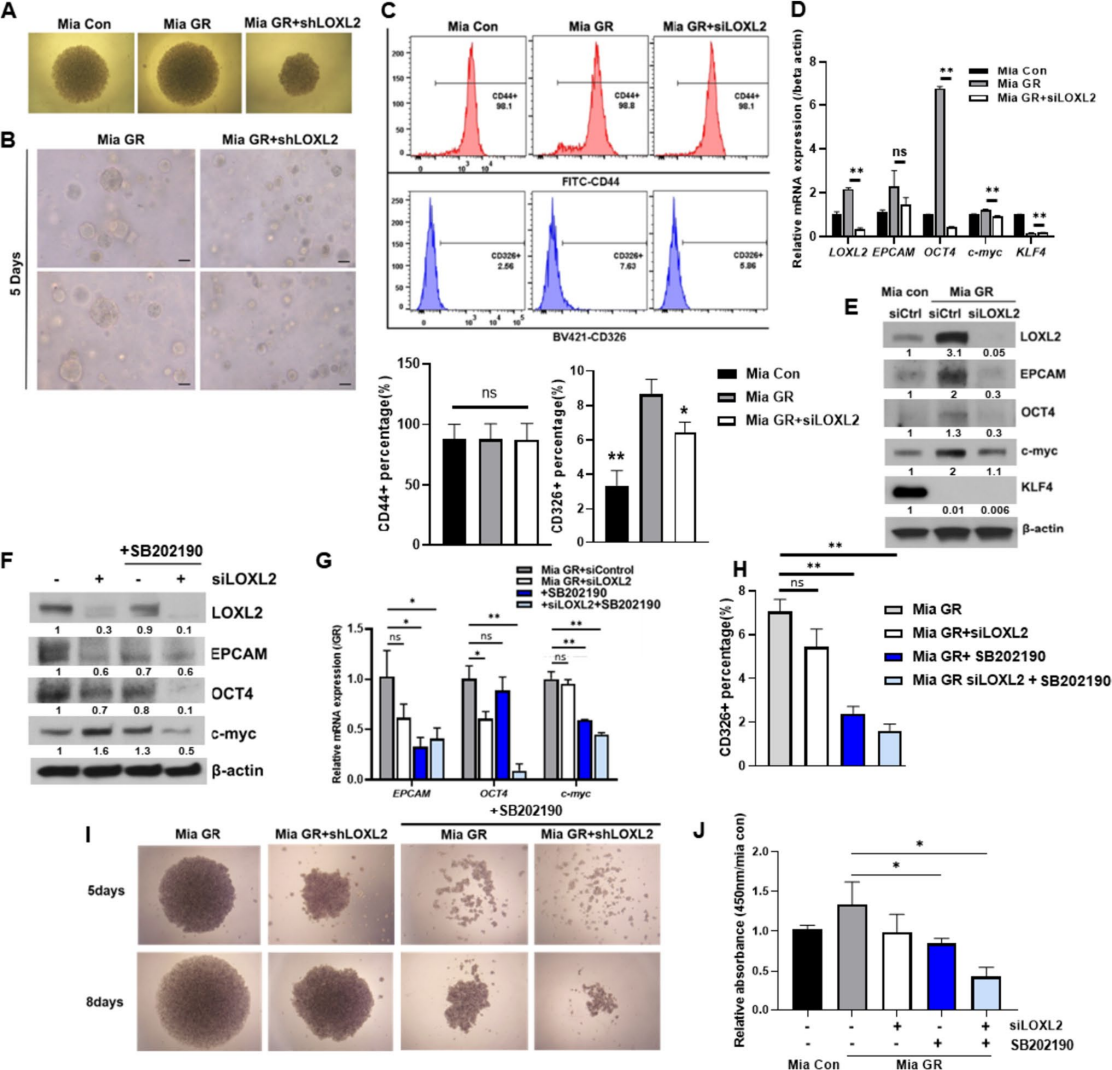
LOXL2 activates the MAPK signaling pathway to modulate cancer stemness of gemcitabine-resistant cells. **A** Microscopic images of spheroid formed Mia Con and Mia GR (control shRNA and LOXL2 shRNA transfected) cells after 8 days in culture (40 × magnification). **B** Image of Mia Con and Mia GR (control, LOXL2 shRNA) in a 3D culture system. Bars = 100 µm. **C** Flow cytometry analysis of pancreatic cancer stem cell surface markers CD44 and EPCAM in Mia Con, Mia GR (control and LOXL2 shRNA). Histograms and bar graphs document the enrichment of CD44 + and CD326 (EPCAM). **D** and **E** Expression of stemness markers in Mia Con and Mia GR (control, LOXL2 shRNA transfected) are analyzed by qPCR D, and immunoblotting (**E)**. **F** and **G** Mia GR cells were treated with SB2190 or/and transfected with siLOXL2. Stemness markers were analyzed by immunoblotting (**F**), and qPCR (**G**). **H** FACS analysis of CD326 + cells in Mia GR cells treated with SB2190 and/or transfected with siLOXL2. **I** Spheroid formation of Mia GR cells. **J** WST-1 based viability assay of Mia GR

Cancer stemness is a key chemoresistant characteristic and contributes to recurrence and drug resistance in pancreatic cancer with high heterogeneity. From mRNA seq results, it was confirmed that the stem cell-related pathway was related according to LOXL2. Therefore, the stemness of the LOXL2 knockdown Mia GR was confirmed through spheroid formation and 3D culture. Therefore, the size of the spheroid was well-formed in Mia GR compared to Mia Con, but did not increase when LOXL2 was knocked down (Fig. 6A). In the 3D culture system, LOXL2 knockdown was confirmed to inhibit the growth of 3D-shaped cells (Fig. 6B). Expression of the CD326 gene and protein (EPCAM), a representative surface marker of cancer stemness, was reduced, and it was found through flow cytometry that CD326 positive cells were decreased by LOXL2 (Fig. 6C). In addition, expression change of “Yamanaka factors,” factors related to cancer stemness, was confirmed, and reduction of Oct4 and c-myc was confirmed through qPCR and western blot (Fig. 6D, E). Conversely, by sorting CD326 + Mia GR, LOXL2 and stemness factors (oct4, c-myc) were overexpressed, through qPCR and western blot (Supple. Fig. 3A, B). Based on the previous mRNA seq results, MAPK was assumed as a sub-factor for regulating LOXL2 stemness and its activity was confirmed by western blot analysis (Fig. S4A, C). In Mia GR, p38 phosphorylation was increased compared to Mia Con, and its activity was decreased by LOXL2 knockdown (Supplementary Fig. S4D). When cells were treated with siLOXL2 and p38 inhibitor SB202190 alone or in combination, the expression of stemness markers was reduced (Fig. 6F, G). Furthermore, the CD326 + cell population and spheroid formation were significantly decreased in the cotreatment environment (Fig. 6H, I). Furthermore, inhibitor and LOXL2 knockdown showed the lowest viability after 8 days of 3D culture (Fig. 6J). Gemcitabine-resistant pancreatic cancer exhibits cancer stemness, which is regulated by LOXL2 and is achieved through the regulation of EPCAM and oct4, c-myc by MAPK signaling.

### LOXL2 promotes tumorigenesis and modulates gemcitabine resistance in xenograft models

**Fig. 7:**
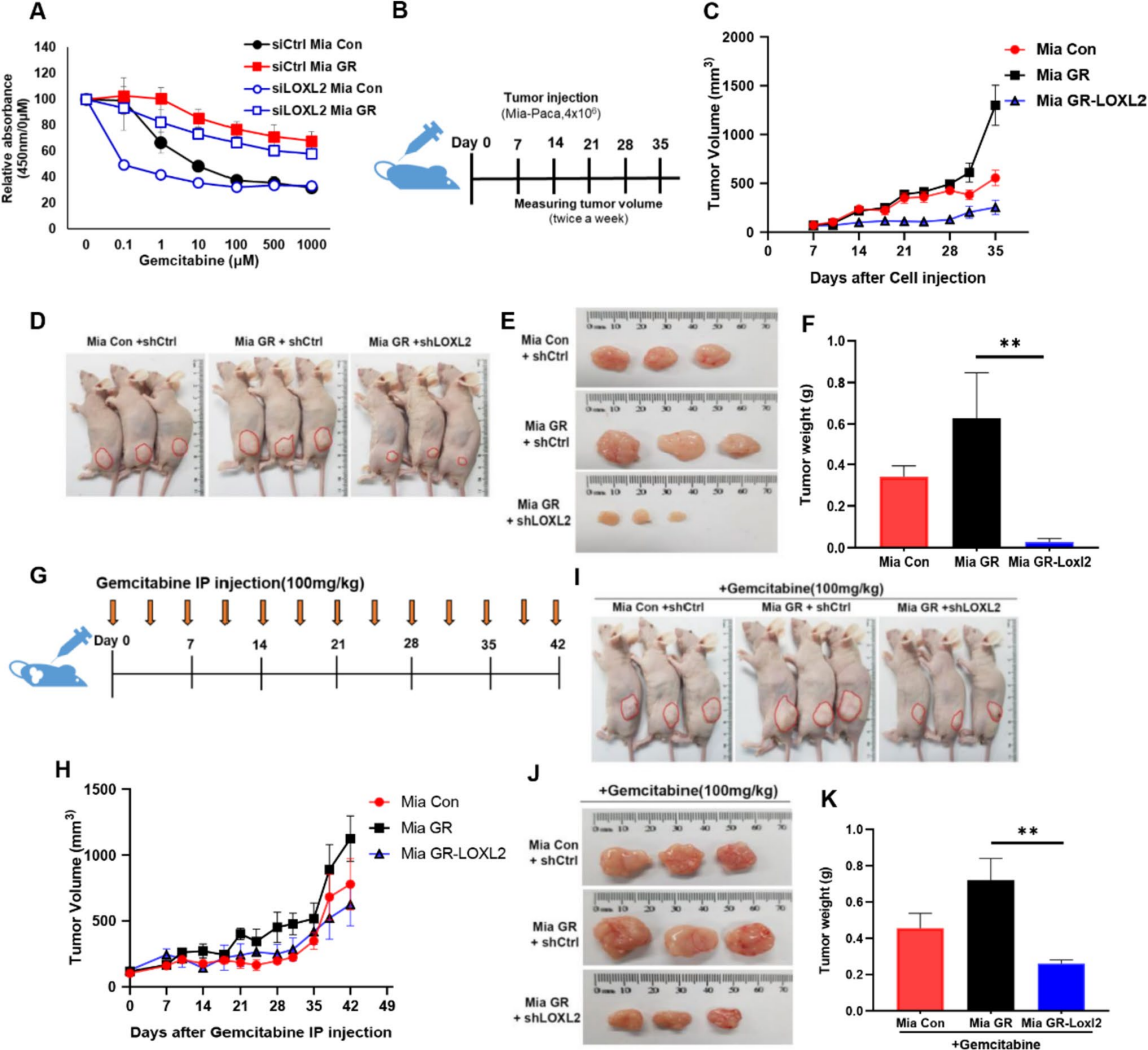
LOXL2 inhibits the tumor growth of gemcitabine-resistant pancreatic cancer cells and increases sensitivity to gemcitabine. **A** WST-1 assay of Mia con and Mia GR (control, LOXL2 siRNA transfection) cells exposed to gemcitabine at different concentrations for 48 h. **B** Scheme of cell administration into the flanks of a nude mouse. **C** Tumor volumes of Mia Con and Mia GR (control, LOXL2 shRNA transfected) administered mouse were measured every 3 days. **D** Representative tumor bearing mouse image. **E** Images of the tumors harvested from mice on day 35. **F** Mouse tumor wet weights on day 35 after tumor inoculation. **G** Scheme of gemcitabine intraperitoneal injection to tumor bearing mouse. (100 mg/kg) **H** Tumor volumes of Mia con and Mia GR (control, LOXL2 shRNA transfected) administered and gemcitabine treated mice were measured every three days. **I** Representative tumor bearing mouse image. **J** Images of tumors harvested from mice on day 42. **K** Tumor wet weights of the mouse on day 42 after gemcitabine I.P. injection

Increased gemcitabine sensitivity by LOXL2 knockdown was observed through the proliferation assay (Fig. 7A). We constructed a xenograft mouse model in Mia GR expecting the EMT process-preferring trait and maintenance of high cancer stemness will affect cancer growth. First, we created a Mia GR cell line that stably knocked down LOXL2 (Fig. S5A, B). It was confirmed that Mia GR tumorigenicity was higher than Mia Con and that LOXL2 knockdown had an inhibitory effect on tumor volume (Figs. 7C and S5C). After 35 days of cell administration, the difference was more clearly defined when weight and size were compared (Fig. 7D, F). After administrating cells in the same way as in the previous xenograft mouse model, the reaction was confirmed by treatment with gemcitabine (Fig. 7G). Tumor growth was slow in mice administered with Mia GR, in which LOXL2 was knocked down (Figs. 7H and S5D). Furthermore, sensitivity to gemcitabine was higher in the LOXL2 knockdown mouse group when volume and weight were sacrificed 6 weeks after drug injection (Fig. 7I and K).

## Discussion

Pancreatic cancer is a fatal multigene-based disease with a high mortality rate. According to 2020 cancer statistics, pancreatic cancer ranks as the seventh leading cause of cancer-related death worldwide. A domestic study conducted in 2021 revealed that pancreatic cancer has a low incidence of only 3.4% but is estimated to have a high mortality rate [37]. Despite the development of many treatments and diagnostic methods, the 5-year survival rate is the lowest among cancer types at 11% [38]. One reason for a poor prognosis is that radiation therapy and chemotherapy can prolong survival or alleviate symptoms but rarely lead to a cure; therefore, resistance to treatment is a major challenge [39]. Studies have shown that even with gemcitabine adjuvant chemotherapy, the 2-year survival rate is only 4%, indicating that most pancreatic cancers exhibit some resistance [40]. Therefore, in this study, our objective was to develop a gemcitabine-resistant pancreatic cancer cell line and confirm the characteristics of the resistance mechanism to improve pancreatic cancer treatment. We found that LOXL2 is highly expressed in gemcitabine-resistant pancreatic cancers compared to the gemcitabine-sensitive by examining both pancreatic patients and pancreatic cancer cells. Moreover, it played a major role in various metabolic mechanisms in gemcitabine-resistant pancreatic cancer than the drug-sensitive cancer. Also, we found that it had a positive correlation with another EMT marker, ZEB1.

LOXL2 is a well-known ECM-related enzyme that catalyzes the formation of collagen crosslinks in the extracellular region. Intracellular LOXL2 also acts as an EMT modulator through the regulation of EMT-inducing transcription factors [41]. Specifically focusing on LOXL2-induced cancer progression, various attempts have recently been made to develop inhibitors [42]. However, a phase II study using the LOXL2 inhibitor simtuzumab for pancreatic cancer did not confirm a significant effect [43]. Regarding LOXL2 and drug resistance, a study published in *Oncotarget* in 2016 reported that it limits intratumoral drug distribution through extracellular enzymatic activity [17]. Besides its functions in EMT and ECM remodeling, we identified a novel role of LOXL2 in stem cell regulation.

Drug-resistant cells undergo many metabolic changes, including upregulation of DNA repair, increased survival signaling and autophagy, activation of drug efflux pumps, and neutralization of ROS [44]. Among them, hypoxic and metabolic stress environments cause pancreatic cancer to show the “Warburg effect,” which activates oncogenes or suppresses tumor suppressor genes and activates glycolysis by increasing glucose uptake [45, 46]. Recently, it was confirmed that translational regulation by HIF1 alpha and MYC ultimately regulates glycolysis in pancreatic cancer [47]. A study revealed that a positive feedback loop exists between LOXL2 and HIF1α, facilitating glycolytic metabolism under hypoxia. Moreover, LOXL2 plays an important role in regulating glucose metabolism in cancer metastasis [48]. In addition, cancer cells’ circulatory systems increase glucose uptake and NF-κB transcriptional activity [49]. Taken together, this study might predict that the induction of HIF-1 by NF-κB activated by glycolysis increases the expression of LOXL2 and induces c-myc to form a feedback loop that regulates glycolysis.

Both NF-κB and ZEB1 are oncogenic transcription factors. ZEB1 plays an important role in EMT regulation, similar to LOXL2. To date, transcriptional regulation of ZEB1 by NF-κB is understood, but its association with LOXL2 is unknown [50]. LOXL2 and ZEB1 are EMT regulators that transform cells from the epithelial type to the mesenchymal type. The EMT process induces drug resistance by increasing drug efflux, slowing cell proliferation, and avoiding apoptosis signaling pathways and immune response [51]. Therefore, overexpression of LOXL2 and ZEB1 was confirmed, and simultaneous regulation of both could effectively inhibit the EMT process in gemcitabine-resistant pancreatic cancer. Therefore, it would be compelling to explore whether the induction of HIF-1 by NF-κB activated by glycolysis would increase not only the expression of LOXL2 but also the expression of ZEB1 with considering the previous study.

Cancer stem cells are immortal tumor cells that can self-renew. They are present in less than 1% of pancreatic cancer but contribute to cancer growth and maintenance, metastasis, and drug resistance. Studies have shown that the potential link between EMT and CSC is considered a key factor in cancer cell plasticity, which acquires cancer drug resistance and transforms cancer cells into malignant cells and vice versa [52]. Although we found that both EPCAM as a surface marker and oct4 and c-myc as Yamanaka factors were confirmed to be regulated by LOXL2, KLF4 as another Yamanaka factor was downregulated regardless of regulation of LOXL2 in Mia GR. Wang et al. [53] demonstrated that gemcitabine suppresses the expression of KLF4 with relation to the chemo-resistant mechanism along with an increase in ZEB1. Therefore, we concluded that stemness could be positively modulated by LOXL2 in gemcitabine-resistant pancreatic cancer cells with the exception of KLF4 expression.

This study investigated the regulatory role of the LOXL2 subfactors in maintaining drug resistance in gemcitabine-resistant pancreatic cancer and aimed to elucidate the induction mechanism leading to LOXL2 overexpression. We confirmed that glucose metabolism was activated in gemcitabine-resistant pancreatic cancer cells, regulating NF-κB signaling. Activated NF-κB not only directly induces transcription by binding to the LOXL2 promoter but also overexpresses ZEB1, resulting in transcriptional regulation of LOXL2 through ZEB1. In gemcitabine-resistant pancreatic cancer, the EMT process was significantly inhibited when ZEB1 and LOXL2 were co-regulated. Furthermore, LOXL2 has also recently been associated with cancer stem cells and plays a regulatory role in a MAPK signal-dependent manner. Therefore, the results revealed an important role for LOXL2 in the resistance to gemcitabine of pancreatic cancer cells and provided an effective therapeutic target to treat pancreatic cancer.

### Supplementary Information

Below is the link to the electronic supplementary material.

Supplementary file1 (TIF 15201 KB)

Supplementary file2 (TIF 15118 KB)

Supplementary file3 (TIF 14779 KB)

Supplementary file4 (TIF 15395 KB)

Supplementary file5 (TIF 14808 KB)

Supplementary file6 (XLSX 9 KB)

Supplementary file7 (XLSX 10 KB)

## Data Availability

The original contributions presented in the study are included in the article/Supplementary Material. Further inquiries can be directed to the corresponding author.
