# Supplementary figures and images for: The role of LOXL2 induced by glucose metabolism-activated NF-κB in maintaining drug resistance through EMT and cancer stemness in gemcitabine-resistant PDAC
Source: J Mol Med (Berl). 2023 Sep 22;101(11):1449–64. doi: 10.1007/s00109-023-02369-6 (PMC10663195; doi:10.1007/s00109-023-02369-6)

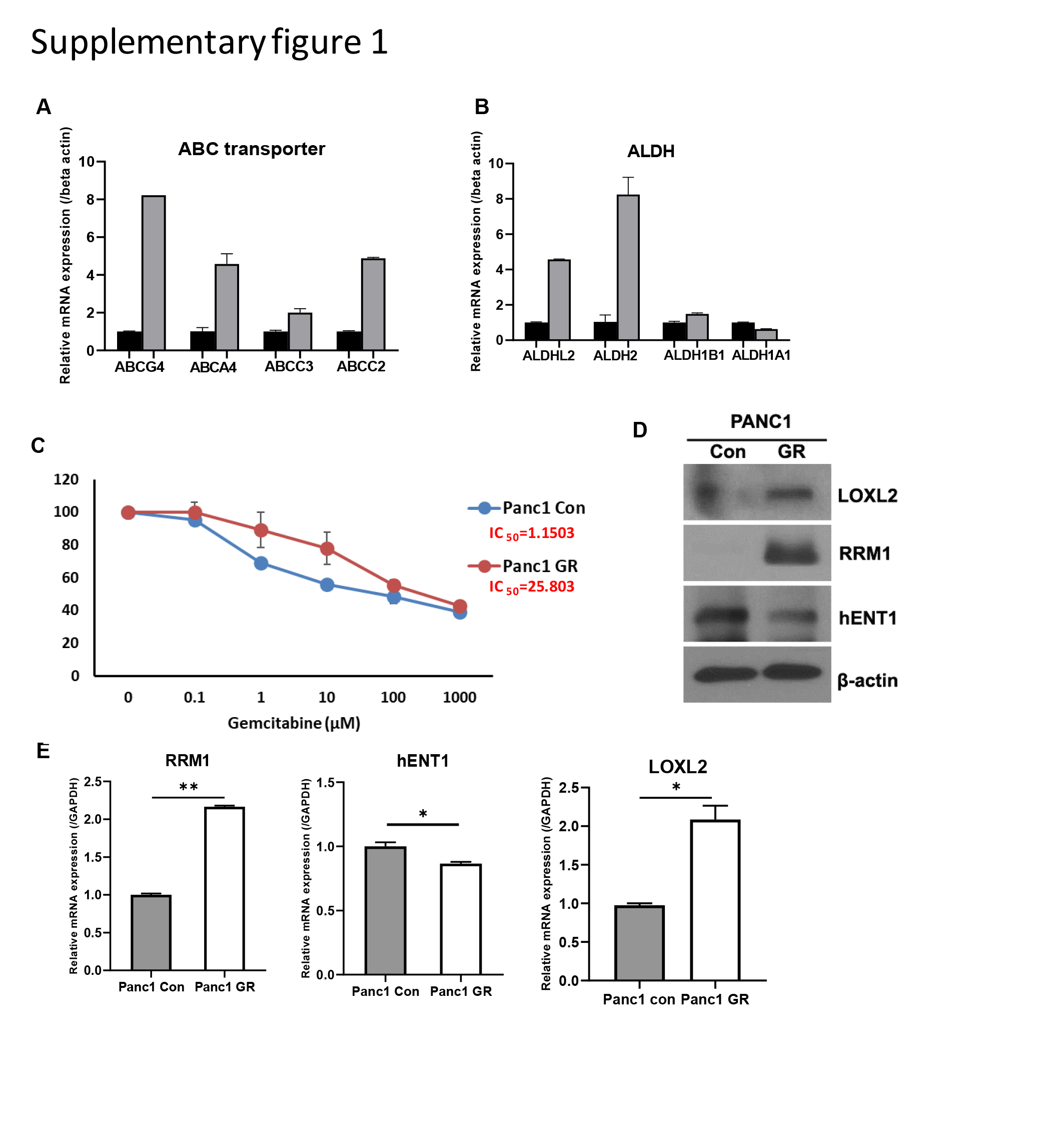

Supplement: Supplementary file 1 — Supplementary file1 (TIF 15201 KB) [file 109_2023_2369_MOESM1_ESM.tif]

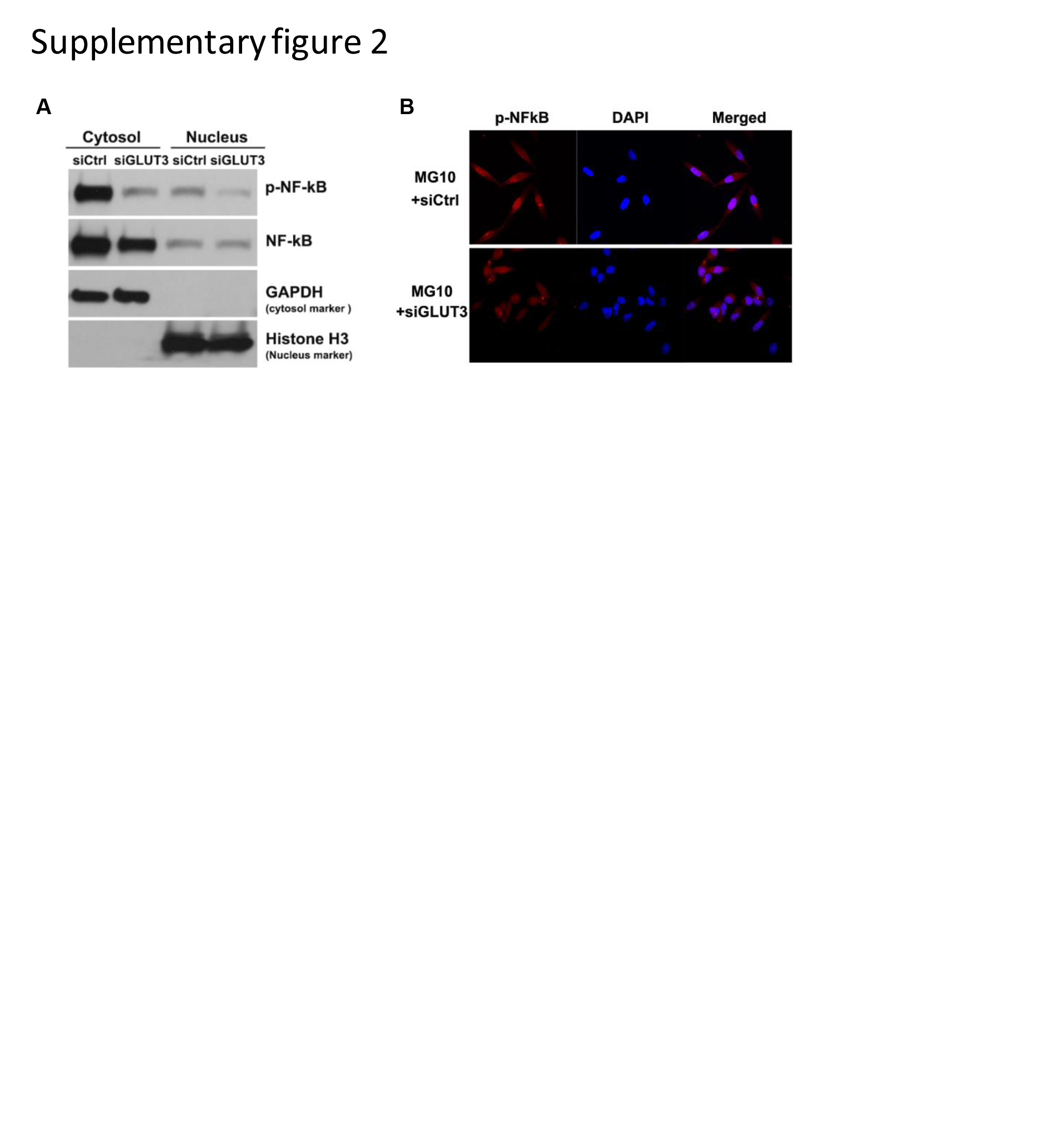

Supplement: Supplementary file 2 — Supplementary file2 (TIF 15118 KB) [file 109_2023_2369_MOESM2_ESM.tif]

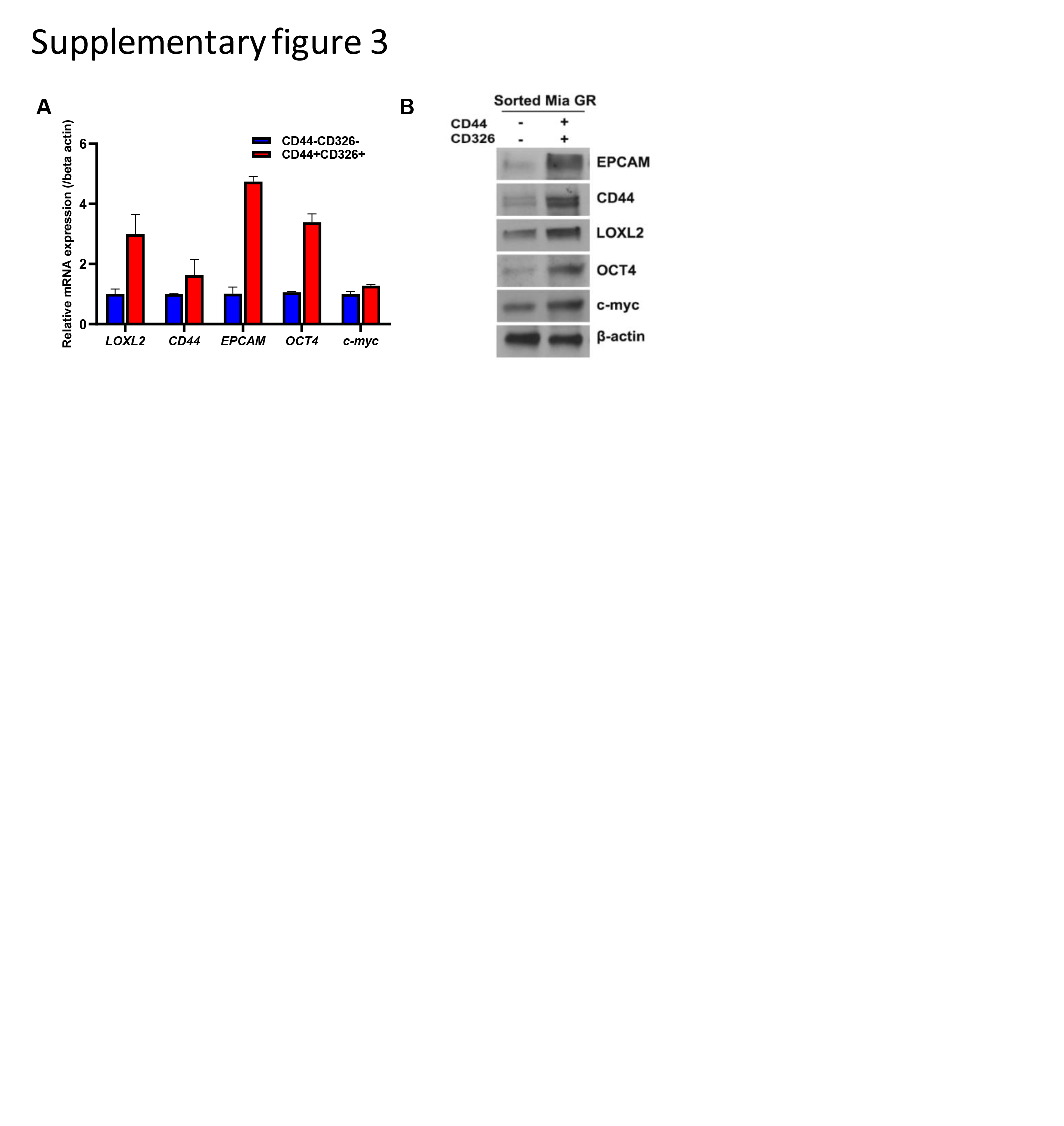

Supplement: Supplementary file 3 — Supplementary file3 (TIF 14779 KB) [file 109_2023_2369_MOESM3_ESM.tif]

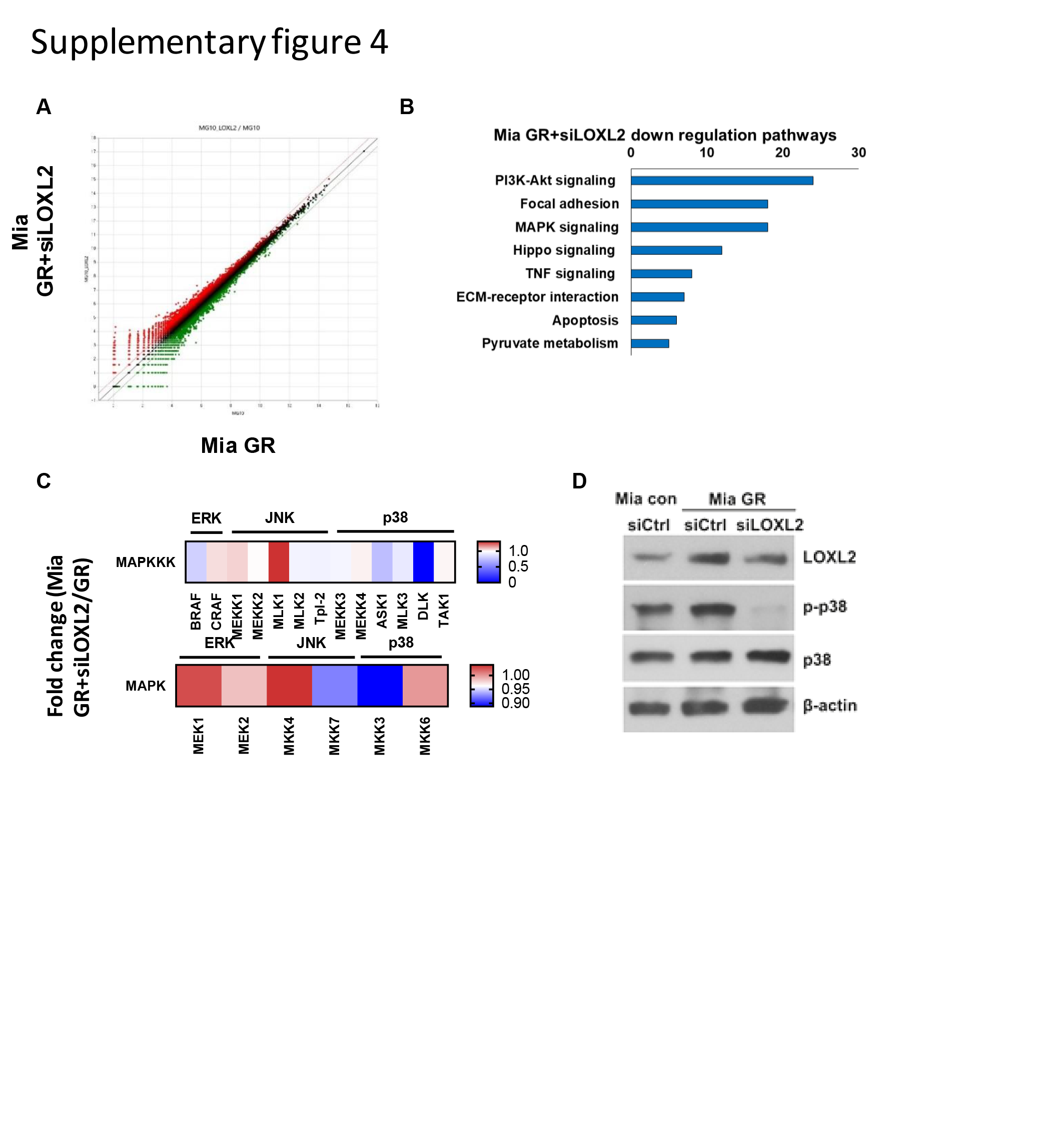

Supplement: Supplementary file 4 — Supplementary file4 (TIF 15395 KB) [file 109_2023_2369_MOESM4_ESM.tif]

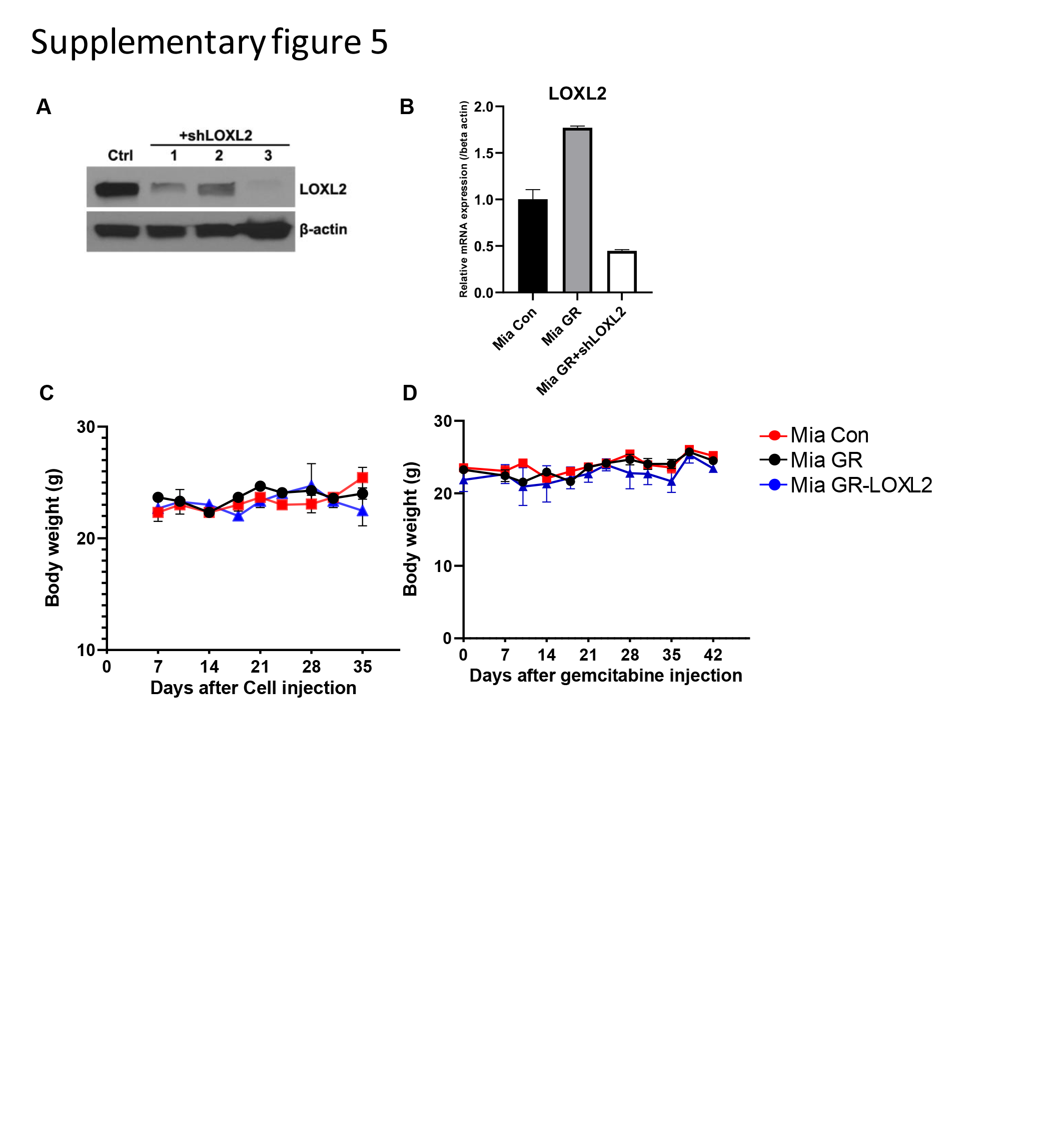

Supplement: Supplementary file 5 — Supplementary file5 (TIF 14808 KB) [file 109_2023_2369_MOESM5_ESM.tif]
